# Broad individual immersion-scattering of respiratory compliance likely substantiates dissimilar breathing mechanics

**DOI:** 10.1038/s41598-021-88925-x

**Published:** 2021-05-03

**Authors:** Olivier Castagna, Guillaume Michoud, Thibaut Prevautel, Antoine Delafargue, Bruno Schmid, Thomas Similowski, Jacques Regnard

**Affiliations:** 1 Underwater Research Team (ERRSO), Military Biomedical Research Institute-IRBA, BP 600, 83800 Toulon Cedex 9, France; 2 Laboratoire Motricité Humaine Expertise Sport Santé (LAMHESS, UPR6312), Nice, France; 3 2e Régiment Etranger de Parachutistes, Calvi, France; 4 Cardiology Department, HIA Ste Anne Toulon, Toulon, France; 5 Pilot Subs, Lanrodec, France; 6 Sorbonne Université, INSERM UMRS1158, Groupe Hospitalier Universitaire APHP Pitié-Salpêtrière Paris, Paris, France; 7 University Bourgogne Franche-Comté, EA3920, University Hospital Besançon, Besançon, France

**Keywords:** Respiration, Experimental models of disease

## Abstract

Head-out water immersion alters respiratory compliance which underpins defining pressure at a “Lung centroid” and the breathing “Static Lung Load”. In diving medicine as in designing dive-breathing devices a single value of lung centroid pressure is presumed as everyone’s standard. On the contrary, we considered that immersed respiratory compliance is disparate among a homogenous adult group (young, healthy, sporty). We wanted to substantiate this ample scattering for two reasons: (i) it may question the European standard used in designing dive-breathing devices; (ii) it may contribute to understand the diverse individual figures of immersed work of breathing. Resting spirometric measurements of lung volumes and the pressure–volume curve of the respiratory system were assessed for 18 subjects in two body positions (upright Up, and supine Sup). Measurements were taken in air (Air) and with subjects immersed up to the sternal notch (Imm). Compliance of the respiratory system (*Crs*) was calculated from pressure–volume curves for each condition. A median 60.45% reduction in *Crs* was recorded between Up-Air and Up-Imm (1.68 vs 0.66 L/kPa), with individual reductions ranging from 16.8 to 82.7%. We hypothesize that the previously disregarded scattering of immersion-reduced respiratory compliance might participate to substantial differences in immersed work of breathing.

## Introduction

Exercise-induced pulmonary edema is somewhat frequent during immersion although more controversial on land^1,2^. Immersion pulmonary edema (IPE) is a potentially life-threatening condition^3–6^ that occurs during swimming, freediving, and SCUBA diving^7^. Impaired cardiovascular conditions, such as hypertension or sensitive arterial pulmonary reactivity, have been recognized to facilitate the onset of IPE^3,8–11^. However, IPE occurrence was also reported early on in fit healthy subjects during substantial exercising effort^4,12,13^. In all cases, the immersion-linked increases in cardiac and pulmonary blood volumes set a foundation for the development of IPE, as they increase pressure in pulmonary vessels^10,14,15^. We observed that after 30 min of controlled immersed exercising with identical dive-breathing devices, (i) the number of ultrasound lung comet tails was substantially higher in some subjects and (ii) the work of breathing was also markedly different between the subjects^16^. In addition, the number of lung comet tails, an indicator of the amount of extravascular lung fluid, correlated with the amount of work of breathing (WOB) and with markers of right heart congestion and of right/left ventricular imbalance^16–18^.

These results raised the question of uncovering the mechanisms likely to underlie such differences in individual figures of WOB. WOB is shaped by various factors among which exercise intensity, body size and thoracic morphometry, gender and age of the subject, potential histological and functional alterations related to lung or thoracic diseases^19^. In our study the subjects were largely a homogenous group as regarding the age range, the sex (only men), the fitness and dive-training, the lack of lung disease as COPD or interstitial fibrosis. In contrast, immersion likely influences WOB through breathing mechanics. The hydrostatic pressure causes increased blood content in the lung vasculature, decreased lung gaseous volumes and impeded breathing movements through imbalance between alveolar and mean external thoracic pressure or “static lung load” (SLL)^14,20–22^ which refers to the “lung centroid pressure” and delineates in turn an additional breathing requirement^22,23^. Inspiratory WOB is greater when swimming than cycling on land at identical ventilatory flow rates^24^. Immersed alterations of breathing mechanics and SLL were firstly assessed through measuring changes in compliance of the respiratory system^21,23,25,26^, but the dispersal of the individual effect has not been analyzed. Differences in individual respiratory compliance would then result in different figures of WOB in similar conditions of immersed exercising amid roughly similar subjects. While the study by Taylor and Morrison depicted scattered individual figures of immersed compliance^23^, a mean value of lung centroid is largely used e.g. to define the European standard EN 14143, suggesting that immersion lowers compliance by a single amount whoever the person. Later a standard SLL of 20 cmH_2_O was proposed as unique reference without alluding possible individual figure deviation^22,27^.

We therefore searched for individual differences in immersion-linked changes in respiratory compliance as one likely determinant of the scaled overloading of ventilatory effort.

The study was designed to assess changes in the pressure–volume relationship of the respiratory system during head-out water immersion. We also examined whether baseline measurements of respiratory compliance in distinct postures on ground could predict immersion-induced changes in respiratory mechanics and provide an estimation for a personal lung centroid.

## Subjects and methods

### Subjects

**Figure 1:**
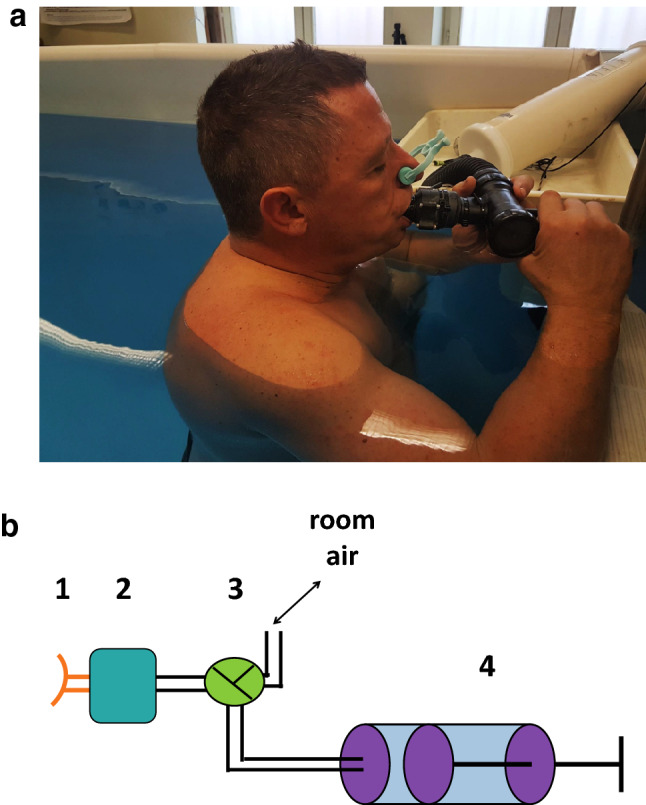
**(a)** A subject during measurement of immersed static respiratory compliance. The subject is kneeling upright and wearing a nose clip. The water comes up to the subject’s the sternal notch. A pressure sensor is integrated in the mouthpiece, which is connected through a pipe to the calibrated syringe to inject or suck up the selected volume. **(b)** Device used to assess pressure and volume. 1, mouthpiece; 2, pressure sensor and transducer; 3, three-way valve; 4, graduated syringe.

Twenty-two men were initially enrolled in the study. All subjects were healthy non-smokers without known pulmonary or cardiovascular diseases or symptoms. Subjects volunteered for enrollment and gave their written informed consent for participating in the study. All the experimental procedures were conducted in line with the declaration of Helsinki, and the study protocol was approved by the local ethics committee (Comité de Protection des Personnes-CPP Sud Méditerranée V, ref 160077). The diver presented on the Fig. 1a, gave his written Informed consent for publication.

### Study design

Body measurements: the thoracic girth (at nipple level) was measured at the end of a normal expiration, and the distances between the sternal notch and xiphoid, and between sternal notch and pubis were also recorded.

Each subject’s breathing was characterized by two measurements: (i) one slow spirometric assessment of lung volumes; (ii) one assessment of overall (thoracic and lung) respiratory static compliance.

The slow vital capacity and the respiratory static compliance were measured in the following four conditions:

– Upright, standing in the air (on land) as control condition (Up-Air),

– Upright, on the knees immersed up to the sternal notch (Up-Imm) in a pool (Fig. 1a),

– Supine, in the air (on land) on a flat stretcher with a cushion under the head, to raise the nostrils above the sternal angle (Sup-Air),

– Supine, immersed up to the sternal notch: the stretcher was lowered into the pool (Sup-Imm) until the subject's sternal notch was at water level.

Tests were performed with the subject wearing a nose clip and breathing through the mouthpiece.

Each subject performed all the tests within a single day at our research unit’s laboratory. The order in which the four conditions were completed was randomly allocated. The temperature in the laboratory was 27 ± 0.5 °C, and subjects wore a loose T-shirt and shorts. In the pool, the water temperature was 34.4 ± 0.5 °C, and subjects wore only trunks.

### Spirometric measurements

A Cosmed Quark PFT Ergo device (Cosmed, Rome, Italy) was used to assess the slow vital capacity (VC), expiratory (ERV) and inspiratory reserve volume (IRV) and tidal volume (VT), according to the guidelines published by the American Thoracic and European Respiratory Societies^28^. Each subject repeated the spirometric maneuver five times in each condition. For each condition, the two extreme values were discarded and the mean of the remaining three was retained.

### Assessing lung-thoracic compliance

Respiratory compliance was determined by replicating the protocols previously described by Taylor and Morrison^23^ to determine the lung centroid position. For each subject, a static pressure–volume curve was constructed in each of the four conditions using values obtained thanks to a specific device. This device was designed in house^16^ and contains a pressure sensor (MPXV70007DP Freescale) with a ± 70-mbar measurement span placed immediately behind a mouthpiece, followed by a flowmeter and a three-way valve (Fig. 1b). Depending on the setting of the three-way valve, the subject, wearing a nose clip, inspires air either from the room or from a syringe filled with a volume selected among the following preset values: 0.2, 0.5, 1, 1.5, 2, 2.5 and 3 L.

For the measurement cycle, the subject initially breathed ambient air freely. At the expiratory end of a quiet tidal volume cycle (i.e. a relaxation volume held without any effort and nominated as *V*_*relax*_), the valve was closed and the subject remained apneic with an open glottis. This relaxed volume was similar to that described by Taylor and Morrison^23^. The mouth pressure in these conditions should be 0 mbar (equal to ambient barometric). If this was not the case, the subject repeated the maneuver. The pulmonary gas volume corresponds to the spontaneous relaxation volume in each condition. In the Up-Air condition, this relaxation volume corresponds to the functional residual capacity (FRC). Once this parameter had been measured, the valve was opened toward the syringe and the subject inspired the preset air volume. As soon as the syringe was empty, the valve was shut down and the subject remained apneic with an open glottis for 4–6 s while the airway pressure was recorded. After each inspired volume, the airway pressure was negative (lower than the ambient atmospheric pressure). The maneuver was performed in a similar manner with the subject starting from *V*_*relax*_ to exhale seven preset volumes (i.e. syringe plunger set and expiration starting with an empty syringe) 0.1; 0.2; 0.5; 1; 1.5; 2; 2.5 and 3 L. As soon as the plunger reached the preset stop, the valve was closed and the airway pressure recorded during the 4–6 s apnea with open glottis. After each expired volume, the pressures recorded were positive (i.e. higher than atmospheric) and reflected the transpulmonary pressure. Subjects were trained to perform the maneuvers so as to reproduce the relaxation volume and the open glottis apnea for each preset volume (i.e. the syringe content or the preset maximal filling allowed). Training required from 30 to 60 min, depending on the subject. Each airway pressure measurement with open glottis at each inspired and expired volume was repeated five times. The two extreme values were discarded before averaging the three remaining values. Data were discarded when the subject could not successfully complete the maneuvers despite several training cycles. These subjects were therefore excluded from the study.

Four curves, one in each condition, were constructed from RV and TLC measurements (both assessed in each condition) for each subject. Curves had an average of 32 ± 8 points depending on the number of volume points achieved. Each curve was fitted using a second square polynomial regression:

$${\text{P}} = {\text{ aV}}^{{2}} {-}{\text{bV}} - {\text{c}}$$

where P is the pressure measured, and V is the gas volume inspired or expired from *V*_*relax*_. The volumes were previously corrected for body temperature and partial pressure of water.

**Figure 2:**
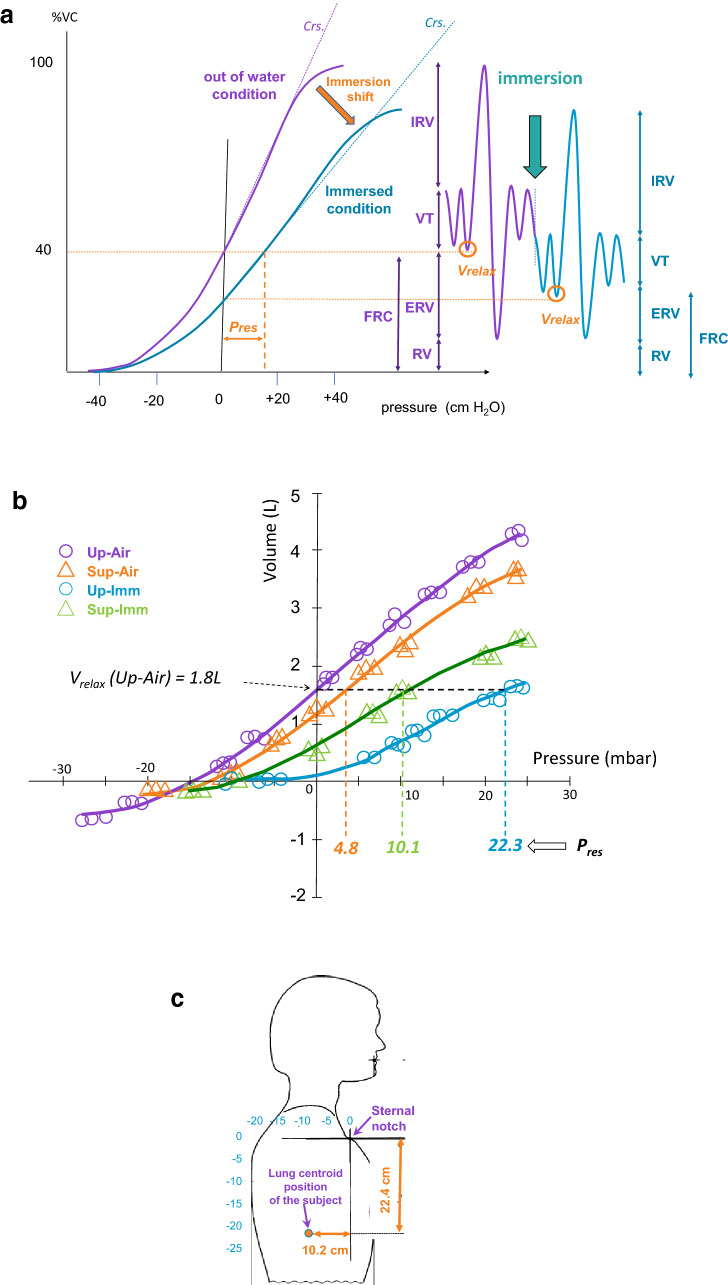
**(a)** Schematic representation of measured and calculated values for the subject on land or immersed. Immersion induced a decrease in *V*_*relax*_, ERV, and FRC, as well as a downward shift of the respiratory compliance curve. *C*_*rs*_ overall compliance of the respiratory system (i.e. combined lung + chest wall), determined from the regression slope for the 1-L increase in volume above V_relax_ plotted against the pressure increase for this 1-L volume; *ERV* expiratory reserve volume; *FRC* functional residual capacity; *IRV* inspiratory reserve volume; *P*_*res*_ restoration pressure assessed as the pressure shift required to resume the Up-Air *V*_*relax*_; *VC* slow vital capacity; *VT* tidal volume; *V*_*relax*_ relaxation volume, is defined as the thoracic volume measured when the respiratory muscles are completely relaxed and the airways are open (Taylor, 1991). **(b)** Pressure–volume curves for a representative subject in the four conditions. In each condition, measurements were repeated three times at each volume (three symbols). For this subject, the *V*_*relax*_ value recorded in the Up-Air condition was 1.8 L. The P_res_ necessary to reach this volume in the Sup-Air, Sup-Imm, and Up-Imm conditions was, respectively, 4.8; 10.1; and 22.3 mbar. Thus, for this subject, the hydrostatic lung centroid pressure is 22.3 mbar in Up-Imm and 10.1 mbar in Sup-Imm. His hydrostatic lung centroid is therefore located 22.4 cm below and 10.2 cm behind his sternal notch. **(c)** Schematic representation of lung centroid position as a function of hydrostatic pressure at the sternal notch. Blue scales show centimeters measured from the sternal notch. Data for the same subject represented in **(b)**.

Each curve was characterized by a value representing compliance of the respiratory system (i.e. combining lung + chest wall, *Crs*), which corresponds to the regression slope for the 1-L increase in volume above *V*_*relax*_ (in the relevant condition) relative to the pressure increase associated with this 1-L volume (*Crs* = ∆V/∆P). A “restoring pressure” value (*P*_*res*_) was also computed for the three alternate conditions, calculated as the difference relative to Air-Up. *P*_*res*_ values were determined as the pressure shift required to return to the Up-Air *V*_*relax*_ value^20^*.* A schematic representation of the measurements performed is presented in Fig. 2a. The three *P*_*res*_ for a representative subject are shown in Fig. 2b, as generated by the shifts in compliance curves for the different conditions.

### Determining the thoracic center of pressure (lung centroid position)

Upright immersion imposes a pressure imbalance between alveolar and mean external thoracic pressure. *P*_*res*_ can be defined as the breathing pressure required to eliminate this imbalance, and is defined by the horizontal (pressure) displacement of immersed compliance curves.

Compliance curves were analyzed using polynomial regression analysis. *P*_*res*_ was determined from isovolume (*V*_*relax*_), pressure–volume curve displacements, measured between control and immersion curves, along the pressure axis. Pressure data corresponded to differential pressures between alveolar pressure (*P*_*alv*_) and skin-applied pressure at sternal notch level. When the pressure unit was chosen as cm of water column (cm H_2_O), the lung centroid position was determined as a point vertically below the sternal notch (during upright immersion), or the sternal plane (during supine immersion). Thus, a two-dimensional estimation of the lung centroid position may be derived and defined as a point within the body relative to a fixed anatomic landmark. The lung centroid position for one specific subject is shown in Fig. 2b, it corresponds to the *P*_*res*_ required to reach the *V*_*relax*_ measured in the control condition (Up-Air).

### Data analysis

Group data are presented as median and first and third quartiles. Mean and standard deviation are also shown. Experimental conditions were statistically compared using Friedman’s rank analysis of variance with post-hoc Dunn’s test. Correlations between variables or changes between conditions were assessed using Spearman’s rank correlation.

## Results

Twenty-two subjects trained to perform slow spirometric assessment of lung volumes and the corresponding airway pressure records, starting from relaxation volume (*V*_*relax*_) in each of the four conditions. Four subjects were unable to achieve satisfactory maneuvers during the immersion conditions, and their data were not included in the analysis presented here. Four static pressure–volume curves were constructed for each of the remaining 18 subjects.

The morphometric characteristics for the 18 subjects retained are presented in Table 1.

**Table 1:** Morphological characteristics of the subjects.

	Age, years	Height, cm	Weight, kg	Chest girth, cm	Sternal length notch-xiphoid, cm	Distance from sternal notch to pubis symphysis cm
Median	35.5	181.5	76.00	98.50	20.00	54.50
Mean	35.94	181.94	76.94	98.39	19.83	54.47
SD	7.63	6.52	6.37	3.82	3.20	1.93

Values for individual subjects are available in “Supplementary Information” files.

Spirometric data (VC, VT, ERV and IRV) for individual subjects are displayed in Table 2.

**Table 2:** Spirometric values measured in each condition.

	In Air Upright				Water Immersion Upright			
	Vital capacity, L	Expiratory reserve volume, L	Tidal volume, L	Inspiratory reserve volume, L	Vital capacity, L	Expiratory reserve volume, L	Tidal volume, L	Inspiratory reserve volume, L
Median	5.19	1.78	0.54	3.02	4.71	0.41	0.74	3.58
Mean	5.13	1.66	0.53	2.94	4.68	0.46	0.73	3.50
SD	0.41	0.32	0.07	0.30	0.37	0.20	0.09	0.50

	In Air Supine				Water Immersion Supine			
	Vital capacity, L	Expiratory reserve volume, L	Tidal volume, L	Inspiratory reserve volume, L	Vital capacity, L	Expiratory reserve volume, L	Tidal volume, L	Inspiratory reserve volume, L
Median	5.03	1.21	0.63	3.21	4.89	0.78	0.69	3.40
Mean	4.96	1.22	0.62	3.12	4.86	0.87	0.67	3.32
SD	0.40	0.35	0.07	0.30	0.37	0.20	0.10	0.50

Individual subject values are available in “Supplementary Information” files.

Median values are shown with mean and standard deviation. Statistical significance was determined using Friedman’s test (4 conditions) and Dunn’s post-hoc test for each comparison between two conditions.

Vital capacity (VC): Friedman p < 0.001; Up-Air vs Sup-Air: NS; Sup-Air vs Sup-Imm: NS; Up-Air vs Sup-Imm: p < 0.001; Sup-Air vs Up-Imm: p < 0.001; Up-Air vs Up-Imm: p < 0.001.

Expiratory reserve volume (ERV): Friedman p < 0.001; Up-Air vs Sup-Air: p < 0.05; Sup-Air vs Sup-Imm: NS; Up-Air vs Sup-Imm: p < 0.01; Sup-Air vs Up-Imm: p < 0.01; Up-Air vs Up-Imm: p < 0.001.

Tidal volume (VT): Friedman p < 0.001; Up-Air vs Sup-Air: p < 0.05; Sup-Air vs Sup-Imm: NS; Up-Air vs Sup-Imm: p < 0.001; Sup-Air vs Up-Imm: p < 0.001; Up-Air vs Up-Imm: p < 0.001.

Inspiratory reserve volume (IRV): Friedman p < 0.001; Up-Air vs Sup-Air: NS; Sup-Air vs Sup-Imm: NS; Up-Air vs Sup-Imm: p < 0.01; Sup-Air vs Up-Imm: NS; Up-Air vs Up-Imm: p < 0.001.

### Immersion-linked changes in spirometric lung volumes

Slow VC was lower in both immersed conditions compared to the reference position, Up-Air (p < 0.001). The expiratory respiratory volume (ERV) decreased progressively from Up-Air to Sup-Air, Sup-Imm and mostly with Up-Imm (p < 0.001), and a narrower individual scattering was noted in immersed conditions. The tidal volume (VT) increased progressively from Up-Air to Sup-Air, Sup-Imm, and Up-Imm (p < 0.001). IRV increased gradually in the same sequence, and individual scattering was broader in the immersed conditions. The breathing frequency decreased similarly in the three other conditions compared to Sup-Air (medians: Up-Air 17.65 min^-1^; Sup-Air 14.06; Sup-Imm 12.99; Up-Imm 13.68 min^-1^; p < 0.001). No significant change in minute ventilation was noted with either condition (medians: Up-Air 9.45 L.min^-1^; Sup-Air 8.50; Sup-Imm 9.95; Up-Imm 8.90 L.min^-1^; NS).

On the whole, both VC and ERV were significantly lower in the two immersed conditions compared to the air conditions (p < 0.001). Conversely, VT and IRC were significantly higher in the immersed conditions than in both air conditions (p < 0.001). The immersion-induced decrease in ERV correlated negatively with the increase in VT (Spearman’s ρ = 0.711; p < 0.001).

### Immersion-linked changes in overall compliance of the respiratory system

A “restoring pressure” (*P*_*res*_) value was computed for the three alternate conditions, each compared to Air-Up. *P*_*res*_ values were determined as the pressure shift required to return to the Up-Air *V*_*relax*_ value^20^*.* An example of the four pressure–volume curves for one subject is shown in Fig. 2b. The *P*_*res*_ for the three conditions compared with Up-Air are indicated. For this subject, the hydrostatic lung centroid pressure is 22.3 mbar in Up-Imm and 10.1 mbar in Sup-Imm. His hydrostatic lung centroid is therefore located 22.4 cm below and 10.2 cm behind his sternal notch (Fig. 2C).

Table 3 displays the individual values for overall respiratory compliance (*C*_*rs*_) in the four conditions and the three *P*_*res*_ values determined for each subject.

**Table 3:** Overall respiratory compliance (*C*_*rs*_) and restoration pressures (*P*_*res*_) measured.

	*C*_*rs*_ (L/kPa)				*P*_*res*_ (kPa)		
	Up-Air	Sup-Air	Sup-Imm	Up-Imm	Sup-Air	Sup-Imm	Up-Imm
Median	1.68	1.12	1.06	0.66	0.46	0.87	1.42
Mean	1.77	1.25	0.97	0.75	0.46	0.87	1.43
SD	0.31	0.60	0.31	0.28	0.17	0.22	0.52

Individual values for each subject are available in “Supplementary Information” files.

Median values are shown with mean and standard deviation. Statistical significance was determined using Friedman’s test (4 conditions) and Dunn’s post-hoc test for comparisons between two conditions.

Compliance of the overall respiratory system (combined lung and chest wall) (*Crs*): Friedman p < 0.001; Up-Air vs Sup-air: NS; Sup-Air vs Sup-Imm: NS; Up-Air vs Sup-Imm: p < 0.01; Sup-Air vs Up-Imm: p < 0.001; Up-Air vs Up-Imm: p < 0.001.

Restoring pressure (P_res_): Friedman p < 0.001; Sup-Imm vs Sup-Air: p < 0.05; Up-Imm vs Sup-Imm: p < 0.01; Up-Imm vs Sup-Air: p < 0.05.

**Figure 3:**
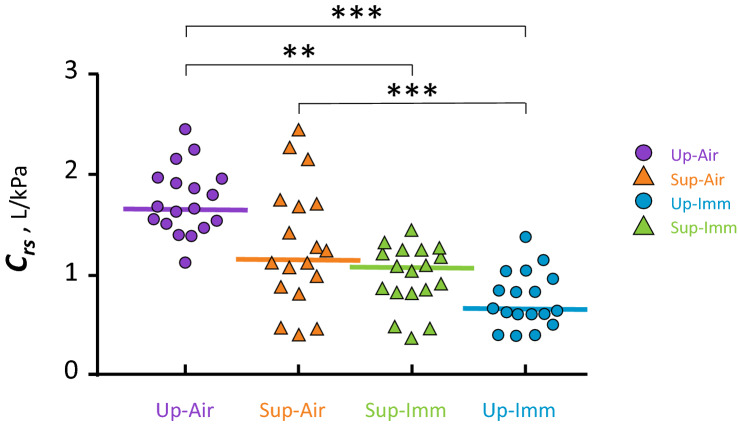
Overall respiratory compliance values (*C*_*rs*_) in the four conditions. The thick lines correspond to median values for the group. Symbols are the same as in Fig. 2b. In most subjects, *C*_*rs*_ decreased substantially between Up-Air and immersed conditions. Statistical analysis used Friedman’s test, and Dunn’s post-hoc tests. **p < 0.01; ***p < 0.001.

**Figure 4:**
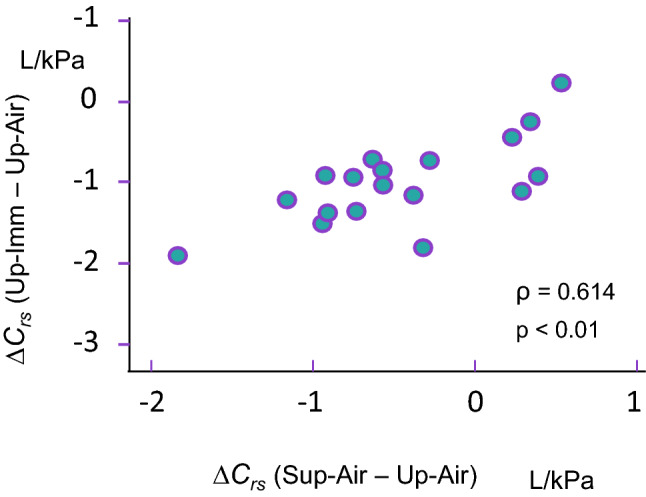
Upright respiratory compliance decreases upon immersion, and the decrease correlates with that measured in air when subjects shifted from an upright to a supine position. Spearman’s rank correlation.

In air, *C*_*rs*_ measured for each subject, in both the upright and supine postures ranged from 0.9–4.0 L/kPa. Changing from Up-Air to Sup-Air reduced *C*_*rs*_ in most subjects, from (median [1st–3rd quartile]) 1.68 [1.52–1.92] L/kPa to 1.17 [0.9–1.7] L/kPa (Friedman p < 0.001; Table 3 and Fig. 3). In line with these changes, the *C*_*rs*_ curves were shifted toward higher pressures for lower volumes, with a *P*_*res*_ of 0.49 [0.35–0.61] kPa. *C*_*rs*_ decreased most extensively between Up-Air: 1.68 [1.52–1.92] L/kPa and Up-Imm: 0.66 [0.59–0.95] L/kPa; p < 0.001, and also to a lesser extent between Sup-Air (1.17 [0.9–1.7] L/kPa) and Up-Imm (0.66 [0.59–0.95] L/kPa, p < 0.001; Fig. 3). The reduction in upright *C*_*rs*_ upon immersion correlated with its reduction upon changing from an upright to a supine position in air (on land) (Fig. 4). No significant correlation was found between the ERV decrease from Up-Air to Sup-Air, nor from Up-Air to Up-Imm or for the decrease in C_rs_ in the corresponding conditions.

**Figure 5:**
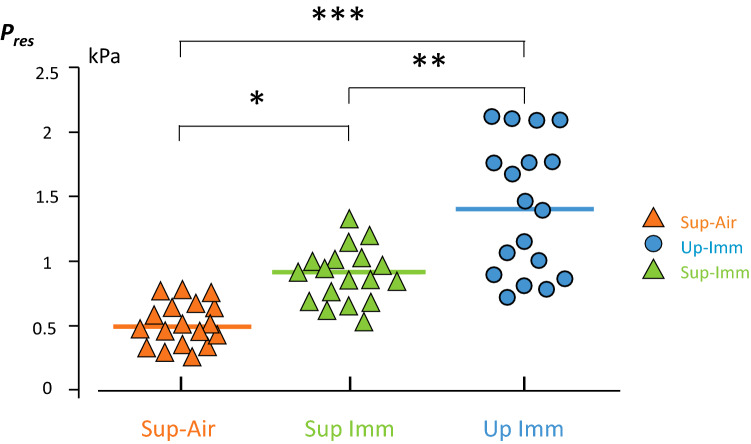
Individual *P*_*res*_ values in the three conditions as compared to Up-Air. The *P*_*res*_ value represents the change in airway pressure required to return to the *V*_*relax*_ value in the Up-Air condition (see Fig. 2a). The thick line corresponds to the median value for the group. Orange triangles: Sup-Air; Green triangles: Sup-Imm; Blue circles: Up-Imm. *p < 0.05; **p < 0.01; ***p < 0.001. Statistical analysis based on Friedman’s test and Dunn’s post-hoc test. Although the median or average *P*_*res*_ value in the Up-Imm condition was almost three-fold that in the Sup-Air condition, individual values were scattered over twice the range in the Up-Imm condition compared to the Sup-Air condition.

The immersed *P*_*res*_ values recorded for *V*_*relax*_ were 1.42 [0.93–1.77] kPa for Up-Imm and 0.88 [0.69–1] kPa for Sup-Imm (Fig. 5). On average, *P*_*res*_ in Up-Imm was about three times that for Sup-Air, with a roughly two-fold increase in scattering for individual values.

No morphometric variable (height, weight, thoracic girth, etc.) was found to correlate with any change in *C*_*rs*_ or *P*_*res*_. The decrease in *C*_*rs*_ upon changing from Up-Air to Up-Imm correlated positively with the decrease in *C*_*rs*_ measured upon changing from Up-Air to Sup-Air (Spearman, ρ = 0.614; p < 0.01; Fig. 5). The *P*_*res*_ for Up-Imm correlated negatively with the decrease in *C*_*rs*_ upon changing from Up-Air to Sup-Air (Spearman, ρ = − 0.589; p < 0.05).

#### Position of the lung centroid

**Figure 6:**
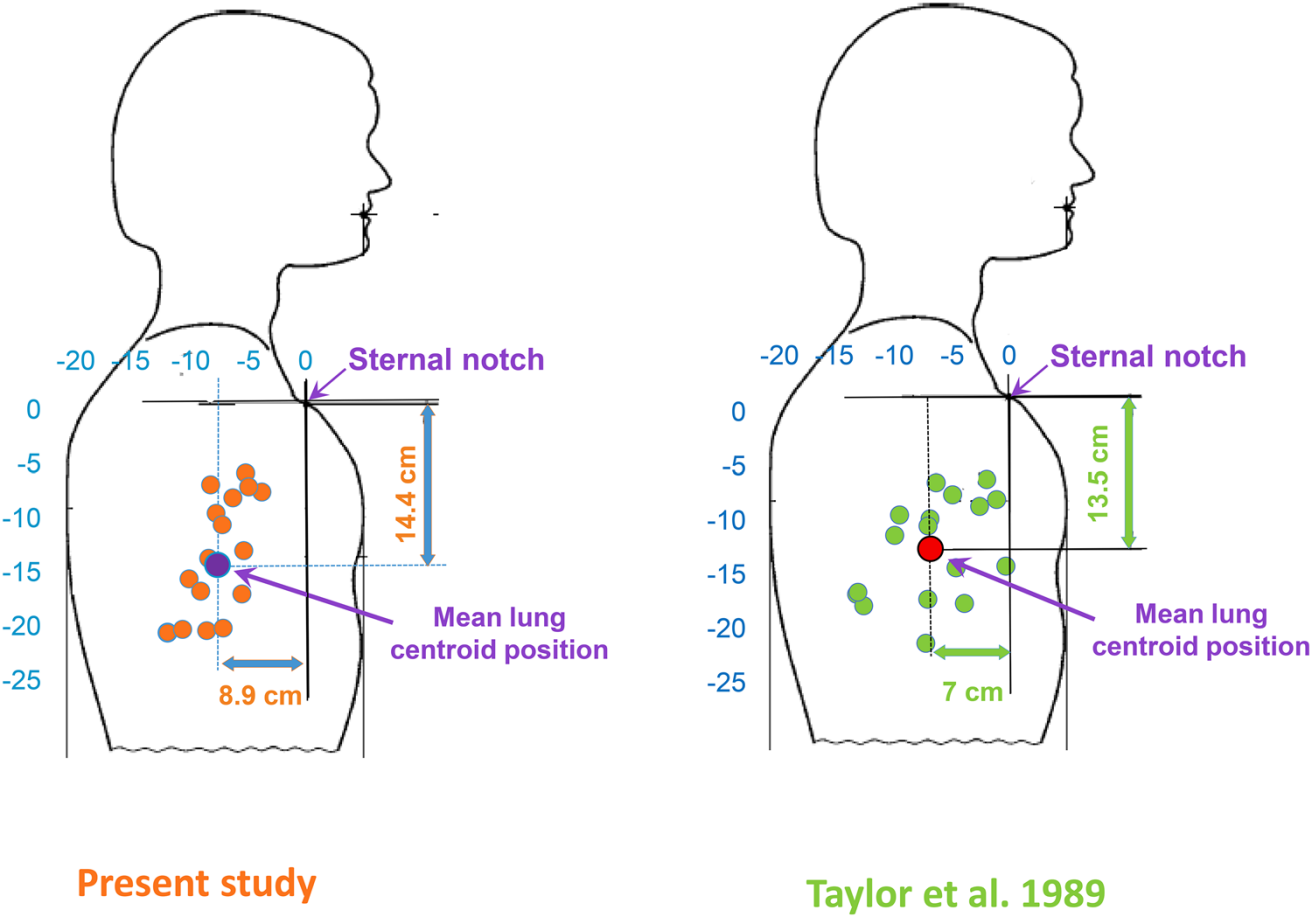
Individual lung centroid positions determined for head-out immersion. Both the shape of the mannequin and its dimensions (mm), as well as the scales (horizontal & vertical) correspond to those in the drawing in the European standard (EN 14143, 2013). These dimensions and shapes must be applied when designing scuba breathing devices. The position of the sternal notch, and the mean theoretical lung centroid position are shown. The left panel displays the results of the present study. Orange circles represent the lung centroid position determined for one subject. Purple circles represent the average lung centroid position based on data for all subjects combined. The right panel shows the results from Taylor and Morisson^20^. Green circles represent individual lung centroid positions for each subject. The red circle is the average lung centroid position determined by combining data from all subjects. This mean lung centroid position (135–70 mm from sternal notch) was chosen as the reference value in the European standard (EN 14143, 2013). The standard thus encompasses a broad scattering of individual values. The overall dispersion of individual values was roughly similar in the two studies, and the slightly different mean values appear coherent.

In each subject, the position of the lung centroid was determined as the distance to the sternal notch on both the antero-posterior axis and the cephalo-rostral axis, based on the *P*_*res*_. On the antero-posterior axis the distance (in cm) was taken as the *P*_*res*_ measured (in mbar) in the Sup-Imm condition. On the cephalo-rostral axis, the distance (in cm) of sternal notch to lung centroid was taken as the *P*_*res*_ (in mbar) measured in the Up-Imm condition. Thus, in the vertical position in left panel of the Fig. 6, the lung centroid ranged between 7.35 and 21.73 cm below the sternal notch (mean 14.44 cm), and the horizontal distance ranged between 5.31 and 13.47 cm posterior to the notch (mean 8.93 cm). Figure 6 displays the individual positions and the average point determined in this study (left panel) and in the former study by Taylor and Morrison (right panel), both relying on the method described by Taylor and Morrison^20^. Detailed individual data are available in supplementary Table 4.

## Discussion

This study assessed immersion-induced changes in spirometric lung volumes and in overall compliance of the respiratory system (*C*_*rs*_) at rest in both upright and supine positions. Individual immersion-linked decreases in *C*_*rs*_ were confirmed, but this impairment was widely scattered and did not correlate with any anthropometric variable. A previously underestimated dispersion of the lung centroid position was also noted.

The immersion-linked reduction in *C*_*rs*_ was associated with increased dispersion of *P*_*res*_ values, i.e. the requirement for higher airway pressures to return to the relaxation volume measured upright in air (FRC). In addition, the decrease in *C*_*rs*_ during upright immersion correlated with the reduction measured upon changing from an upright to a supine posture in air (on land) (Fig. 4). The decreases in *C*_*rs*_ also correlated with decreases in ERV, which inversely correlated with increases in VT.

### Changes in lung volumes

The lowering of ERV between a standing and a supine position in air reflects a decrease in residual functional capacity^29,30^. Reductions in VC and ERV upon immersion have been repeatedly described^20,21,25,31–34^. Both lying supine in air and immersion cause a cephalad displacement of the diaphragm and reduce lung volumes^21,35^. Our observation of an increased IRV in parallel to the decrease in ERV upon immersion is in line with results presented by Paton and Sand^31^ and Dahlbäck et al.^34^. Paton and Sand^31^ also described a larger ERV decrease for vertical immersion than for lying in air. In the present study, the increase in VT was less marked, but immersion caused a greater reduction in ERV.

Finally, it has to be considered that in the supine position both in air and immersed, part of the decrease in lung gas volume is induced by the intrathoracic pooling of the blood volume^15,36,37^.

### Changes in C_rs_

The changes in *C*_*rs*_ measured in this study were in line with previous reports. Jarrett^25^ described relaxation pressure–volume curves that were shifted along the pressure axis in the same ranking order (with a similar magnitude): Up-Air, Sup-Air, Sup-Imm, and Up-Imm. Taylor and Morrison^23^ established the transpulmonary pressure–volume relationship in 17 immersed subjects in upright and prone positions, and compared them with upright and supine conditions in air. The results revealed a substantial intersubject variability for pressure–volume curves related to either immersion or posture. The results presented here indicated a similarly extensive scattering of *C*_*rs*_ and also revealed a correlation between postural changes in air and air-to-immersion changes.

*C*_*rs*_ changes combine alterations in compliance for both lung tissue and the chest wall. Firstly, immersion increases the lung blood volume, causing a substantial decrease in parenchymal compliance^14,15,34,38^. In patients with congestive heart failure, a rapid 22% decrease in lung compliance was observed when they changed their position from sitting upright to lying down^39^. Secondly, lung compliance also decreases simply when the gaseous lung volume is reduced. In healthy young subjects, changing from sitting upright to a supine position shifted the pressure–volume curve to a 5–8-cmH_2_O lower transpulmonary pressure, and decreased dynamic lung compliance when the breathing rate increased (an effect known as frequency dependence)^40^. This reduction in compliance was effectively related to lung volume rather than to the change in position as it was reproduced when the sitting subjects breathed to their supine end-expiratory volume, i.e. using a reduced lung volume. In the present study, this mechanism was probably implemented during the Sup-Air and Sup-Imm conditions, where reduced lung gaseous volumes were measured compared to the Up-Air condition. In anesthetized children, the *C*_*rs*_ measured at FRC is reported to be lower in a lateral than in a supine position, due to the lower inspiratory capacity in the lateral position. This postural difference was extended with both increasing age and height^41^. Thirdly, in air, chest wall compliance is reported to be lower in a supine position compared to upright^42^. Ingimarssson et al.^41^ indicated that the chest wall accounted for 33% of the total elastance of the respiratory system in a lateral position compared to just 12% in a supine position. During water immersion, the hydrostatic pressure exerts a similar restrictive effect that also increases chest wall elastance.

Thus, in our subjects, the three foregoing components probably combined to shift the pressure–volume curves and reduce the overall compliance of the respiratory system. Firstly, the lung blood content was increased in a supine position in air, and further with immersion. Secondly, lung volume was reduced both in the air after upright to supine tilting and in the same positions, when changing from air to water immersion. Thirdly, the hydrostatic restriction of the trunk further increased chest wall elastance. The contribution of each component was probably different for each subject^21,32^, and remains impossible to circumscribe based solely on the measurements gathered for this study. However, the strong correlation between the individual decreases in ERV and *C*_*rs*_ values suggest a substantial role for the reduction in lung volume both in a supine position in air and upon immersion as compared to the Up-Air condition. In pigs, redistribution of lung aeration markedly improved lung compliance and FRC independently from lung recruitment^43^. Furthermore, immersion makes pulmonary blood flow distribution more even than in air, but also enlarges VA**’**/Q**’** disparities relative to the increasingly uneven distribution of alveolar ventilation and to airway gas trapping^34,35,43^. Strong indirect evidence indicates that both lying down and being immersed lead to similar changes in cardiac and pulmonary blood volume^37^, as each maneuver led to very comparable values for lung transfer of carbon monoxide as well as for baroreflex stimulation and cardiac autonomic control settings. A similar increase in lung blood volume could thus contribute to the correlation of *C*_*rs*_ changes between the two maneuvers (Fig. 4).

Thus, in our subjects, on the one hand, the reduction in *C*_*rs*_ in parallel with the reduced ERV and FRC were probably due to impaired lung aeration either as a result of changing from an upright to a supine position in air or following immersion. The extent of impairments was different in each condition. On the other hand, the alteration of chest wall compliance also contributed differently in each condition. The lack of statistical relationship between the reductions in ERV and *C*_*rs*_ might therefore be caused by distinct individual contributions of lung volume and chest wall compliance to *C*_*rs*_ changes.

The significant negative correlation between changes in ERV and VT upon immersion results in a lower ERV decrease when VT increases in response to either lying in air or being immersed. A VT increase probably augments the gas content of the lung, regardless of ERV, and would thus increase lung compliance according to the aforementioned mechanism. The graded VT increase also suggests a parallel graduated increase in inspiratory effort^44^. This type of effort would depend on both the extent of the increase in VT and the magnitude of *P*_*res*_. During immersion, positive pressure breathing increases ERV and reduces breathing discomfort^31,45,46^ probably as a result of increased gaseous lung volume and lung compliance as mentioned above, and also of the reduced *P*_*res*_^35,47^. In addition, during immersed exercising, a lower WOB was measured with positive compared to negative pressure breathing^16^. The immersion-linked decrease in lung compliance increases the elastic WOB^31^. In intubated patients, raising them from supine to a 45° semi-seated position increased breathing comfort, allowed PEEP assistance to be reduced and relieved the respiratory muscles^48^. In the present study, the different individual changes in *C*_*rs*_ and *P*_*res*_ caused by immersion at rest appeared to occur alongside changes in distribution of lung volumes and VT. *P*_*res*_ figures in immersion conditions amounted to 5.2–21.3 mbar, and thus stood roughly within the range of transrespiratory system linked to hydrostatic pressure or SLL, which encompass both the “true” transpulmonary pressure—i.e. the pressure difference across the airway opening and the pleura—and the effect of hydrostatic pressure on thoracic wall compliance^35,49^. Here, very similar positions and hydrostatic pressures with regard to anatomical landmarks produced marked individual differences in *P*_*res*_, which defines the lung centroid position^23^. The lung centroid positions measured in the present study are shown in Fig. 6 alongside the reference value used to define rebreathing devices (European standard EN 14413). The implication of the broad scattering of individual lung centroid positions must be considered. When a rebreathing device designed according to a single standard of hydrostatic imbalance is used, the additional transthoracic negative pressure required from different divers could range between 3 and 15 mbar, as their lung centroids are located at between 7 and 21 cm from the sternal notch. This would result in broad scattering of the range of the ensuing elastic WOB^50^, thus fostering the development of IPE in individuals required to exert higher breathing efforts.

It has been assessed that small-sized lungs or a low airway diameter with respect to lung volume (dysanapsis ratio) increase the resistive WOB^50,51^ and thus correlate with susceptibility to IPE, for example in women^11,52^. These anatomical features cause an airflow impedance that is already included in the transpulmonary pressure, and would be of little importance at rest^49^. Up to now, no evidence that the dysanapsis ratio is involved in pulmonary gas trapping and pulmonary barotrauma has been presented. Conversely, an immersion-linked decrease in overall respiratory compliance that is already noticeable at rest would lead to a high strain when high ventilatory flows are required such as during exercising and the dysanapsis ratio, or small-sized lungs might then further boost the strain and increase the breathing effort.

### Implications

This study might have two implications. Firstly, relating to the *P*_*res*_ value used to define the position of the lung centroid pressure in the upright and prone postures^23^. In Taylor and Morrison’s previous work^20^, the average values of the hydrostatic lung centroid pressure were 13.6 cmH_2_O (upright) and 7 cmH_2_O (prone), or 13.3 and 6.86 mbar, respectively. These values have become the standards used when designing rebreathing devices (± 0.5 mbar) (European EN 14143). However, these values are means, even though individual *P*_*res*_ values were found to be broadly scattered in both the initial^23^ and the present studies (Fig. 6). As a result, using a single setting on the breathing device might lead to different individual breathing efforts, substantiating distinct susceptibilities to pulmonary edema^53^. Before different individual susceptibility to IPE was suspected and the wide spread of WOB in similar immersed exercising conditions was reported, different individual dyspnea sensations were also described in similar external breathing resistance conditions^47^.

We therefore propose that standard reference values such as that used in the European standard (EN 14143) should be revised to more precisely represent the meaning of lung centroid pressure and to carefully take into account the wide dispersion of individual values around the average value.

In addition, if the role of *C*_*rs*_ and *P*_*res*_ values in determining breathing effort during exercise were to be confirmed, the consistency between immersion-linked changes in the mechanics of breathing and changes from an upright to a supine position on land might allow a simple test of the individual tendency to higher immersed WOB through assessment of the decrease in *C*_*rs*_, and increase in VT during a postural change on dry ground.

### Limitations

Our study has several limitations.

The interlinked responses to changes in lung gas volume and changes in chest wall compliance could not be separated, even though the distinction might be instrumental in determining degrees of individual susceptibility to an excess WOB in immersed or diving conditions.

Although a higher WOB during swimming than cycling at similar ventilatory flow rates seems to supply immersion-impeded breathing upon exercise^24^, it remains to be confirmed that the decrease in *C*_*rs*_ caused by either lying down or immersion correlates with the WOB during immersed exercising. Retrospective analysis should indicate whether decreases in *C*_*rs*_ are more pronounced in swimmers or divers who have suffered from IPE or recurrent IPE compared to controls who did not experience IPE in similar conditions.

Our study did not closely consider intersubject differences in morphometrics, age and gender which influence the WOB besides the compliance of respiratory system. However aging influences WOB through histological and functional alterations of both lung parenchyma and thoracic wall which directly influence the compliance of the respiratory system.

This study involved only men subjects, as it was firstly related to an odd occurrence of IPE in healthy military divers. Gender-linked anatomical and mechanical thoracic differences might blur the results or entangle their interpretation. However similar studies have to be conducted with women, owing to both these respiratory functional differences and to the seemingly higher proneness of women to IPE^11,50,51^.

## Conclusions

The results presented in this article confirm the main known effects of immersion on breathing patterns and respiratory mechanics—leading to reduced functional residual capacity, expiratory reserve volume, and total respiratory compliance, with an increased tidal volume. Immersion was found to cause a broadly scattered reduction of individual respiratory compliance, with up to three-fold decreased values in some subjects compared to others. Accordingly, considering a standard position of the “lung centroid” when designing breathing equipment could be improper as this barycenter of immersed airway pressure appears to be largely individual.

Since respiratory compliance underlies the elastic WOB, its scattered immersion-linked lowering would likely contribute among other factors to the individual spreading of WOB upon performing similar exercises in immersed conditions. The results also highlighted that relevant information about individual immersion-linked effects on respiratory compliance might be obtained by assessing changes in respiratory compliance in air upon switching from an upright to a supine position.

## Supplementary Information

Supplementary Tables.

## Abbreviations

C_rs_: Overall compliance of the respiratory system
C_th_: Thoracic compliance (lung + chest wall) calculated between V_relax_ (Up-Air) to 1 L higher
ERV: Expiratory reserve volume
FRC: Functional residual capacity
IPE: Immersion pulmonary edema
IRV: Inspiratory reserve volume
NPB: Negative pressure breathing
PPB: Positive pressure breathing
P_res_: Restoration pressure
SLL: Static lung load
Sup-Air: Supine in air condition
Sup-Imm: Supine immersed
Up-Air: Upright in air condition
Up-Imm: Upright immersed
VC: Slow vital capacity
VT: Tidal volume
V_relax_: Relaxation volume i.e. FRC upright in the air and in other conditions, lung volume with open glottis and atmospheric airway pressure
WOB: Work of breathing
