# Supplementary material for: Broad individual immersion-scattering of respiratory compliance likely substantiates dissimilar breathing mechanics
Source: Sci Rep. 2021 May 3;11:9434. doi: 10.1038/s41598-021-88925-x (PMC8093428; doi:10.1038/s41598-021-88925-x)
Supplement: Supplementary file 1 — Supplementary Tables. [file 41598_2021_88925_MOESM1_ESM.docx]

**Broad individual immersion-scattering of respiratory compliance likely substantiates dissimilar breathing mechanics**

**Authors**:

Olivier Castagna*^1,2^, Guillaume Michoud^3^, Thibaut Prevautel^4^, Antoine Delafargue^5^, Bruno Schmid^1^, Thomas Similowski^7^, Jacques Regnard^8^

**Affiliations**

1. Underwater research team – ERRSO, Military biomedical research institute-IRBA, BP 600, 83800 Toulon, France
2. Laboratoire Motricité Humaine Expertise Sport Santé (LAMHESS, UPR6312), Nice France
3. 2e Regiment Etranger de Parachutistes, Calvi, France
4. Cardiology Department HIA Ste Anne Toulon France, France
5. Pilot Subs, Lanrodec, France
6. Sorbonne Université, INSERM UMRS1158, Groupe Hospitalier Universitaire APHP Pitié-Salpétrière, Paris, France
7. Université Bourgogne Franche-Comté, EA3920 University Hospital, Besançon, France.

*Corresponding author

Pr. Olivier. Castagna, MD, PhD

Underwater research team (ERRSO). Military biomedical research institute (IRBA)

BP 600 – 83800 TOULON cedex 9, France

E mail: [castagna.olivier@gmail.com](mailto:castagna.olivier@gmail.com)

Tel: (+33) 6 77 115 661

Fax: (+33) 4 83 162 859

**Supplementary Dataset**

**Tables**

**Table 1**. Morphological characteristics of study subjects.

| Subject  identifier | Age  *years* | Height  *cm* | Weight  *kg* | Chest  girth  *cm* | Sternal  length  notch-xyphoid  *cm* | Distance from  sternal notch to  pubis symphysis  *cm* |
| --- | --- | --- | --- | --- | --- | --- |
| 1 | 45 | 177 | 78 | 97 | 20 | 53 |
| 2 | 27 | 195 | 75 | 97 | 21 | 54 |
| 3 | 45 | 174 | 68 | 97 | 18 | 55 |
| 4 | 40 | 181 | 82 | 97 | 20 | 52 |
| 5 | 35 | 185 | 75 | 95 | 19 | 54 |
| 6 | 36 | 181 | 75 | 98 | 19 | 54 |
| 7 | 31 | 188 | 72 | 100 | 20 | 55 |
| 8 | 42 | 180 | 86 | 100 | 20 | 52 |
| 9 | 32 | 185 | 77 | 103 | 22 | 56 |
| 10 | 42 | 173 | 73 | 99 | 20 | 57 |
| 11 | 25 | 182 | 83 | 100 | 19 | 58 |
| 12 | 26 | 172 | 86 | 103 | 21 | 55 |
| 13 | 26 | 177 | 68 | 95 | 18 | 54 |
| 14 | 30 | 187 | 89 | 91 | 21 | 58 |
| 15 | 42 | 188 | 73 | 102 | 21 | 55 |
| 16 | 46 | 174 | 80 | 104 | 18 | 55 |
| 17 | 31 | 187 | 68 | 91 | 19 | 52 |
| 18 | 46 | 189 | 77 | 102 | 21 | 52 |
|  |  |  |  |  |  |  |
| Median | 35.5 | 181.5 | 76.00 | 98.50 | 20.00 | 54.50 |
| Mean | 35.94 | 181.94 | 76.94 | 98.39 | 19.83 | 54.47 |
| S.D. | 7.63 | 6.52 | 6.37 | 3.82 | 3.20 | 1.93 |

**Table 2**. Spirometric values for all subjects in each condition.

| Subject  identifier | In Air  Upright | | | | Water Immersion  Upright | | | |
| --- | --- | --- | --- | --- | --- | --- | --- | --- |
|  | Vital Capacity | Expiratory Reserve Volume | Tidal Volume | Inspiratory Reserve Volume | Vital Capacity | Expiratory Reserve Volume | Tidal Volume | Inspiratory Reserve Volume |
|  | L | L | L | L | L | L | L | L |
| 1 | 4.74 | 2.20 | 0.44 | 2.10 | 4.38 | 0.64 | 0.58 | 3.16 |
| 2 | 5.23 | 1.79 | 0.60 | 2.84 | 4.85 | 0.51 | 0.80 | 3.55 |
| 3 | 5.10 | 1.45 | 0.55 | 3.10 | 4.67 | 0.68 | 0.79 | 3.20 |
| 4 | 4.84 | 1.13 | 0.60 | 3.11 | 4.43 | 0.72 | 0.84 | 2.88 |
| 5 | 5.15 | 1.87 | 0.41 | 2.87 | 4.66 | 0.31 | 0.58 | 3.77 |
| 6 | 5.92 | 2.22 | 0.56 | 3.14 | 5.36 | 0.17 | 0.75 | 4.44 |
| 7 | 5.56 | 1.91 | 0.59 | 3.06 | 5.08 | 0.11 | 0.77 | 4.19 |
| 8 | 4.82 | 1.55 | 0.57 | 2.70 | 4.38 | 0.41 | 0.78 | 3.20 |
| 9 | 5.36 | 1.79 | 0.51 | 3.06 | 4.90 | 0.38 | 0.72 | 3.80 |
| 10 | 4.42 | 1.27 | 0.59 | 2.56 | 4.01 | 0.51 | 0.83 | 2.66 |
| 11 | 5.37 | 1.76 | 0.49 | 3.12 | 4.84 | 0.25 | 0.68 | 3.92 |
| 12 | 4.78 | 1.44 | 0.52 | 2.82 | 4.33 | 0.32 | 0.72 | 3.29 |
| 13 | 5.06 | 1.45 | 0.52 | 3.09 | 4.64 | 0.42 | 0.73 | 3.49 |
| 14 | 5.53 | 1.45 | 0.59 | 3.49 | 5.13 | 0.64 | 0.84 | 3.65 |
| 15 | 5.27 | 1.79 | 0.58 | 2.90 | 4.90 | 0.35 | 0.80 | 3.75 |
| 16 | 4.37 | 1.11 | 0.51 | 2.75 | 3.97 | 0.74 | 0.72 | 2.52 |
| 17 | 5.50 | 1.82 | 0.43 | 3.25 | 4.96 | 0.75 | 0.59 | 3.62 |
| 18 | 5.23 | 1.85 | 0.40 | 2.98 | 4.75 | 0.35 | 0.55 | 3.84 |
|  |  |  |  |  |  |  |  |  |
| **Median** 1st-3d q | **5.19** 4.8-5.4 | **1.78** 1.5-1.8 | **0.54** 0.5-0.6 | **3.02** 2.8-3.1 | **4.71** 4.4-4.9 | **0.41** 0.3-0.6 | **0.74** 0.7-0.8 | **3.58** 3.2-3.8 |
| *Mean* | ***5.13*** | ***1.66*** | ***0.53*** | ***2.94*** | ***4.68*** | ***0.46*** | ***0.73*** | ***3.50*** |
| *s.d.* | *0.41* | *0.32* | *0.07* | *0.30* | *0.37* | *0.20* | *0.09* | *0.50* |

| Subject  identifier | In Air  Supine | | | | Water Immersion  Supine | | | |
| --- | --- | --- | --- | --- | --- | --- | --- | --- |
|  | Vital Capacity | Expiratory Reserve Volume | Tidal Volume | Inspiratory Reserve Volume | Vital Capacity | Expiratory Reserve Volume | Tidal Volume | Inspiratory Reserve Volume |
|  | L | L | L | L | L | L | L | L |
| 1 | 4.57 | 1.69 | 0.53 | 2.27 | 4.58 | 1.04 | 0.56 | 2.98 |
| 2 | 5.07 | 1.48 | 0.70 | 3.04 | 5.02 | 0.89 | 0.77 | 3.36 |
| 3 | 4.94 | 0.92 | 0.65 | 3.26 | 4.84 | 1.10 | 0.72 | 3.02 |
| 4 | 4.68 | 0.71 | 0.69 | 3.28 | 4.62 | 1.18 | 0.74 | 2.70 |
| 5 | 4.99 | 1.55 | 0.50 | 3.03 | 4.85 | 0.74 | 0.54 | 3.57 |
| 6 | 5.74 | 1.95 | 0.66 | 3.33 | 5.55 | 0.64 | 0.67 | 4.24 |
| 7 | 5.42 | 1.55 | 0.68 | 3.25 | 5.26 | 0.57 | 0.67 | 4.02 |
| 8 | 4.66 | 0.95 | 0.67 | 2.86 | 4.55 | 0.75 | 0.76 | 3.04 |
| 9 | 5.19 | 1.28 | 0.61 | 3.26 | 5.08 | 0.75 | 0.70 | 3.63 |
| 10 | 4.27 | 0.91 | 0.69 | 2.74 | 4.20 | 0.96 | 0.76 | 2.47 |
| 11 | 5.20 | 1.21 | 0.58 | 3.29 | 5.02 | 0.65 | 0.62 | 3.74 |
| 12 | 4.63 | 0.90 | 0.60 | 3.01 | 4.53 | 0.76 | 0.66 | 3.11 |
| 13 | 4.87 | 1.19 | 0.61 | 3.28 | 4.81 | 0.76 | 0.72 | 3.33 |
| 14 | 5.34 | 0.90 | 0.67 | 3.67 | 5.31 | 1.03 | 0.83 | 3.45 |
| 15 | 5.11 | 1.20 | 0.68 | 3.08 | 5.07 | 0.74 | 0.77 | 3.56 |
| 16 | 4.22 | 0.74 | 0.60 | 2.92 | 4.14 | 1.14 | 0.65 | 2.35 |
| 17 | 5.32 | 1.57 | 0.52 | 3.42 | 5.14 | 1.21 | 0.50 | 3.43 |
| 18 | 5.09 | 1.27 | 0.49 | 3.17 | 4.92 | 0.79 | 0.46 | 3.66 |
|  |  |  |  |  |  |  |  |  |
| **Median** 1st-3d q | **5.03**  17-5.2 | **1.21** 0.9-1.5 | **0.63** 0.6-0.7 | **3.21** 3.0-3.3 | **4.89** 4.6-5.1 | **0.78** 0.75-1.1 | **0.69** 0.6-0.75 | **3.40** 3.0-3.6 |
| *Mean* | *4.96* | *1.22* | *0.62* | *3.12* | *4.86* | *0.87* | *0.67* | *3.32* |
| *S.D.* | *0.40* | *0.35* | *0.07* | *0.30* | *0.37* | *0.20* | *0.10* | *0.50* |

Median values are shown with 1st and 3rd quartiles. Statistical significance was determined using Friedman’s test (4 conditions) and Dunn’s post-hoc test for each comparison between 2 conditions.

Vital capacity (VC): Friedman p < 0.001; Up-Air vs Sup-air: NS; Sup-Air vs Sup-Imm: NS; Up-Air vs Sup-Imm: p < 0.001; Sup-Air vs Up-Imm: p < 0.001; Up-Air vs Up-Imm: p < 0.001.

Expiratory reserve volume (ERV): Friedman p < 0.001; Up-Air vs Sup-Air: p < 0.05; Sup-Air vs Sup-Imm: NS; Up-Air vs Sup-Imm: p < 0.01; Sup-Air vs Up-Imm: p < 0.01; Up-Air vs Up-Imm: p < 0.001.

Tidal volume (VT): Friedman p < 0.001; Up-Air vs Sup-Air:p < 0.05; Sup-Air vs Sup-Imm: NS; Up-Air vs Sup-Imm: p < 0.001; Sup-Air vs Up-Imm: p < 0.001; Up-Air vs Up-Imm: p < 0.001.

Inspiratory reserve volume (IRV): Friedman p < 0.001; Up-Air vs Sup-Air: NS; Sup-Air vs Sup-Imm: NS; Up-Air vs Sup-Imm: p < 0.01; Sup-Air vs Up-Imm: NS; Up-Air vs Up-Imm: p < 0.001.

**Table 3**. Overall respiratory compliance (*C_rs_*) and restoration pressures (*P_res_*) for individual subjects.

| Subject  identifier | ***C_rs_***  *(L/kPa)* | | | | ***P_res_*** *(kPa)* | | |
| --- | --- | --- | --- | --- | --- | --- | --- |
|  | Up-Air | Sup-Air | Sup-Imm | Up-Imm | Sup-Air | Sup-Imm | Up-Imm |
| 1 | 1.97 | 1.06 | 0.82 | 0.58 | 0.74 | 0.83 | 2.09 |
| 2 | 1.66 | 1.13 | 1.22 | 1.38 | 0.25 | 0.63 | 0.72 |
| 3 | 1.92 | 0.97 | 0.47 | 0.41 | 0.61 | 1.19 | 2.10 |
| 4 | 1.51 | 0.87 | 0.90 | 0.83 | 0.43 | 0.94 | 0.79 |
| 5 | 1.80 | 1.41 | 1.08 | 0.65 | 0.61 | 1.00 | 1.77 |
| 6 | 1.68 | 1.10 | 1.04 | 0.83 | 0.47 | 0.83 | 1.15 |
| 7 | 2.15 | 2.43 | 1.17 | 1.03 | 0.27 | 1.13 | 1.68 |
| 8 | 1.96 | 1.23 | 1.19 | 0.61 | 0.56 | 0.95 | 1.46 |
| 9 | 2.45 | 2.13 | 1.24 | 0.64 | 0.49 | 0.98 | 2.11 |
| 10 | 1.63 | 0.45 | 0.46 | 0.41 | 0.74 | 1.02 | 1.77 |
| 11 | 1.67 | 1.10 | 1.08 | 0.67 | 0.45 | 0.67 | 1.78 |
| 12 | 2.25 | 0.39 | 0.36 | 0.39 | 0.75 | 1.32 | 2.13 |
| 13 | 1.54 | 0.80 | 0.84 | 0.60 | 0.43 | 0.90 | 1.06 |
| 14 | 1.56 | 1.26 | 0.86 | 0.85 | 0.31 | 0.74 | 0.90 |
| 15 | 1.39 | 1.73 | 1.26 | 1.15 | 0.41 | 0.52 | 0.86 |
| 16 | 1.40 | 0.46 | 0.81 | 0.50 | 0.65 | 0.67 | 1.39 |
| 17 | 1.47 | 1.70 | 1.43 | 1.03 | 0.33 | 0.60 | 0.81 |
| 18 | 1.86 | 2.25 | 1.31 | 0.95 | 0.31 | 0.83 | 1.01 |
|  |  |  |  |  |  |  |  |
| **Median** | **1.68**  1.52-1.95 | **1.12**  0.9-1.69 | **1.06**  0.83-1.21 | **0.66**  0.59-0.93 | **0.46**  0.35-0.61 | **0.87**  0.69-1.0 | **1.42**  0.93-1.78 |
| Mean | 1.77 | 1.25 | 0.97 | 0.75 | 0.46 | 0.87 | 1.43 |
| S.D. | 0.31 | 0.60 | 0.31 | 0.28 | 0.17 | 0.22 | 0.52 |

Median values are shown with 1st and 3rd quartiles. Statistical significance was determined using Friedman’s test (4 conditions) and Dunn’s post-hoc test for comparisons between 2 conditions.

Compliance of the global respiratory system (combined lung and chest wall) (*Crs*): Friedman p < 0.001; Up-Air vs Sup-air: NS; Sup-Air vs Sup-Imm: NS; Up-Air vs Sup-Imm: p < 0.01; Sup-Air vs Up-Imm: p < 0.001; Up-Air vs Up-Imm: p < 0.001.

Restoring pressure (Pres): Friedman p < 0.001; Sup-Imm vs Sup-Air: p < 0.05; Up-Imm vs Sup-Imm: p < 0.01; Up-Imm vs Sup-Air: p < 0.001.

**Table 4** . Individual position of the lung centroid in the present study and in Taylor& Morisson (1989)

|  | Present study | |  | Taylor et al. | |
| --- | --- | --- | --- | --- | --- |
| subject | vertical (cm) | horizontal (cm) | subject | vertical (cm) | horizontal (cm) |
| 1 | 21.33 | 8.47 | 1 | 9.7 | 2.5 |
| 2 | 7.35 | 6.43 | 2 | 8.8 | 5 |
| 3 | 21.43 | 12.14 | 3 | 18.3 | 7.1 |
| 4 | 8.06 | 9.59 | 4 | 11.1 | 6.9 |
| 5 | 18.06 | 10.20 | 5 | 15.5 | 4.8 |
| 6 | 11.73 | 8.47 | 6 | 10.7 | 9.5 |
| 7 | 11.91 | 11.53 | 7 | 22.4 | 7.2 |
| 8 | 14.90 | 9.69 | 8 | 15.4 | 0.5 |
| 9 | 21.53 | 10.00 | 9 | 9.6 | 1.2 |
| 10 | 18.06 | 10.41 | 10 | 18.6 | 12.8 |
| 11 | 18.16 | 6.84 | 11 | 11.5 | 7 |
| 12 | 21.73 | 13.47 | 12 | 18.8 | 4 |
| 13 | 10.82 | 9.18 | 13 | 14.8 | 14.8 |
| 14 | 9.18 | 7.55 | 14 | 7.8 | 6.5 |
| 15 | 8.78 | 5.31 | 15 | 12.6 | 10 |
| 16 | 14.18 | 6.84 | 16 | 7.5 | 2 |
| 17 | 8.27 | 6.12 | 17 | 17.7 | 13.1 |
| 18 | 10.31 | 8.47 |  |  |  |
|  |  |  |  |  |  |
| mean | 14.44 | 8.93 |  | 13.58 | 6.76 |
| median | 14.18 | 8.83 |  | 12.60 | 6.90 |
| min/max | 7.3 / 21.7 | 5.3 / 13.5 |  | 7.5 / 22.5 | 2 / 14.8 |

All values are expressed using the sternal notch as a reference point. These values were obtained from individual *P_res_* (mbar) values converted to H_2_O cm

No significant differences between results from the present study and Taylor & Morisson (1989) were observed.

Vertically, p= 0.789 (unpaired Student’s t test)

Horizontally, p= 0.0632 (unpaired Student’s t test)
